# Capturing fine-scale travel behaviors: a comparative analysis between personal activity location measurement system (PALMS) and travel diary

**DOI:** 10.1186/s12942-018-0161-9

**Published:** 2018-12-03

**Authors:** Mingyu Kang, Anne V. Moudon, Philip M. Hurvitz, Brian E. Saelens

**Affiliations:** 1 Urban Form Lab, Department of Urban Design and Planning, University of Washington, 1107 NE 45th St, Suite 535, Seattle, WA 98195 USA; 2 Department of Pediatrics, Seattle Children’s Research Institute, University of Washington, 2001 Eighth Avenue, Suite 400, Seattle, WA 98121 USA

**Keywords:** Automated algorithm, GIS, GPS, Accelerometer, Trips, Places

## Abstract

**Background:**

Device-collected data from GPS and accelerometers for identifying active travel behaviors have dramatically changed research methods in transportation planning and public health. Automated algorithms have helped researchers to process large datasets with likely fewer errors than found in other collection methods (e.g., self-report travel diary). In this study, we compared travel modes identified by a commonly used automated algorithm (PALMS) that integrates GPS and accelerometer data with those obtained from travel diary estimates.

**Methods:**

Sixty participants, who made 2100 trips during seven consecutive days of data collection, were selected from among the baseline sample of a project examining the travel behavior impact of a new light rail system in the greater Seattle, WA (USA) area. GPS point level analyses were first conducted to compare trip/place and travel mode detection results using contingency tables. Trip level analyses were then performed to investigate the effect of proportions of time overlap between travel logs and device-collected data on agreement rates. Global performance (with all subjects’ data combined) and subject-level performance of the algorithm were compared at the trip level.

**Results:**

At the GPS point level, the overall agreement rate of travel mode detection was 77.4% between PALMS and the travel diary. The agreement rate for vehicular trip detection (84.5%) was higher than for bicycling (53.5%) and walking (58.2%). At the trip level, the global performance and subject-level performance of the PALMS algorithm were 46.4% and 42.4%, respectively. Vehicular trip detection showed highest agreement rates in all analyses. Study participants’ primary travel mode and car ownership were significantly related to the subject-level mode agreement rates.

**Conclusions:**

The PALMS algorithm showed moderate identification power at the GPS point level. However, trip level analyses found lower agreement rates between PALMS and travel diary data, especially for active transportation. Testing different PALMS parameter settings may serve to improve the detection of active travel and help expand PALMS’s applicability in geographically different urbanized areas with a variety of travel modes.

## Background

Identifying and understanding human location and movement is a crucial part of transportation planning, public health, and health geography research [1]. Previous studies have shown that people who walk or bike for transportation are more likely to meet public health recommendations by accumulating more physical activity [2, 3]. Since the 1970s, governmental planning agencies have relied on travel survey data in order to create transportation models [4–7], including identifying origins and destinations of people’s reported movements, travel modes, and related activities. More recently, a growing interest in active transportation (primarily walking and bicycling) on the part of public health [2, 3, 8, 9], has lead researchers to obtain more objectively measured mobility data through the use of such devices as global positioning system (GPS) data loggers and accelerometers to inform transportation models. However, computational algorithms are required to process the massive quantities of GPS and accelerometer data generated in these studies [5, 8, 10–13], and to date, limited work has evaluated these algorithms to understand the details on how they quantify fine-scale travel behaviors.

Self-reported travel diaries are one of the most common instruments for obtaining data on people’s locations and movements [14, 15]. Traditionally, travel diaries have been considered as the comprehensive source of information for travel behaviors [16]. This self-report approach allows for the collection of data on trip purposes, departure and arrival times, travel mode, and related respondent characteristics including age, sex, race/ethnicity, education level, income, health status, etc. Unfortunately, self-reported travel surveys are a burdensome, leading to low compliance and inaccuracy. The resultant data are susceptible to human errors such as recall or social desirability bias [17]. Common problems include missed trips, incomplete entries, and misreported time stamps and travel behavior characteristics [18]. Nevertheless, important behavioral data such as purpose of travel, visited place names, addresses, and certain travel modes cannot be obtained without study participants’ direct input [19]. In addition, in many instances, the primary measurement method for travel behaviors remains self-reported because of cost and feasibility [14, 15].

Combined use of individual-based GPS and accelerometer devices to measure location, speed, and physical activity levels has provided new opportunities to characterize travel behaviors at fine spatial and temporal scales [19, 20]. During the last few years, the use of these types of devices to obtain objectively measured travel behaviors has taken a salient role in active transportation and physical activity studies [21, 22]. Although not entirely error-free, these techniques are considered to provide more objective and accurate measures of travel behavior than the traditional methods that rely on active respondent reporting [23]. As passive data collectors, GPS and accelerometer devices reduce study participant burden and likely enhance data quality [24]. Also, GPS data offer information on traveler’s route choice and corresponding speed, which have been difficult to collect through travel survey methods [5].

However, processing massive GPS and accelerometer data sets to reconstruct mobility patterns in terms of trips, trip origins and destinations, and travel mode, requires robust computational power, sophisticated algorithms, and expertise in data management skills [10]. In addition, algorithms often require expert user input for specification of parameter settings for data processing.

Personal Activity Location Measurement System (PALMS) is a web-accessible system enabling the development of travel behavior and physical activity variables from device data [25, 26]. Its main purpose is to merge and process time-stamped data from devices such as GPS data loggers, accelerometers, and heart-rate monitors. PALMS was developed by the Center for Wireless and Population Health Systems, University of California, San Diego. PALMS identifies trips and places, and it categorizes trips into three travel modes: walking, bicycling, or vehicular trips. In addition to processing raw time-stamped data, PALMS can aggregate the data into more manageable sets by day, participant, or event.

Besides PALMS, several algorithms have been developed to measure and quantify visited locations, travel behaviors, and physical activities by using GPS and accelerometer data. Some of these algorithms rely solely on the geographical coordinates information collected via standalone GPS data loggers or mobile phones [1, 10, 27]. These algorithms use frequency, density, and speed information from the spatially-referenced data to detect and classify activity locations and trips. Other algorithms including PALMS use GPS with accelerometer data to identify fine-scale travel behaviors such as walking [12, 28, 29].

The performance of the PALMS algorithm was evaluated in previous studies [8, 25], and results showed moderate agreement rates for travel modes (agreement using SenseCam [25]: 65.3–93.4%; agreement using travel logs [8]: 74.2–89.8%). The performance of other algorithms varied considerably across studies, to include: the percentage of correctly identified trips that were recorded in a travel diary (78.9–86.0%) [5]; the proportion of correctly identified stop locations (92.3%) [10]; the proportion of locations for which a GPS-interview results match was found (50–100%) [20]; the proportion of GPS data time that correctly identified activity location and trip occurrence by comparing with recall interview results (95.8%) [30].

In the travel diary framework, respondents are instructed to record characteristics of individual trips, rather than at fixed time intervals. Therefore, many previous studies assessing specific algorithms used trips as the unit of analysis by connecting consecutive GPS data points sharing a single identified travel mode [5, 31–34]. Since people perceive their travel behaviors based on the trip unit, this approach is appropriate.

However, since PALMS produces output data as GPS points with corresponding trip/place information at every time interval (e.g., minute), previous studies assessed the PALMS algorithm using individual GPS point records as the unit of analysis. PALMS was assessed in previous studies by comparing its classification of trips by mode with SenseCam images from forty adult cyclists [25], and travel logs from two research assistants [8]. In these studies, the PALMS algorithm was evaluated through a collective measure; person-level GPS data were coalesced into a single data set and analyzed in aggregate. Data were aggregated because the sample size was too small to conduct subject-level analyses, and socioeconomic and other demographics information was not obtained from study participants.

The objective of the present study was to compare PALMS travel behavior information with that of travel diaries to address the strengths and weaknesses of the PALMS automated algorithm. According to reviews of GPS data processing methods and studies of the reliability of wearable activity trackers [35, 36], no other study assessing PALMS or other algorithms conducted the analyses at both GPS and trip levels using the same data sources. In the present study, we first combined data from all subjects to assess PALMS global performance, and second, we evaluated PALMS performance at the participant level to assess individual performance. Lastly, to investigate reasons for possible discrepancies across different levels of analyses, we looked into the socioeconomic characteristics of study participants.

## Methods

### Data development

#### Participants and Sampling

Participant data came from the Travel Assessment and Community (TRAC) project, which recruited > 700 baseline participants within the greater Seattle, WA (USA) area between July 2008 and July 2009. The purpose of TRAC was to investigate effects of a new light rail transit (LRT) system on people’s travel behaviors in two follow-up data collection efforts [12, 37].

For this study, we sampled 60 subjects from baseline TRAC participants. Because the analysis focused on travel mode identification, we conducted a stratified random sample of participants to ensure adequate coverage of the less common travel modes (e.g., transit, walking). Participants were first sorted into groups of drivers (58.1% of the full cohort), transit users (3.8%), or walkers (30.9%), based on the travel mode that each person reported most frequently in his or her travel diary (the remaining 7.2% of participants used bicycle, motorcycle, or taxi for their primary travel mode). From among each of the 3 groups, we randomly selected 20 participants.

Subjects in the final sample were 52.4 years old on average, 46.7% were male, 73.3% were white non-Hispanic, 65% completed a bachelor’s or higher degree, 48.3% had full time jobs, 48.3% reported an annual household income ≤ $50 k USD, 43.3% were married/partnered. The average household size was 2.1 with 0.4 children. Households had an average of 1.1 cars, and 45% lived in single-family housing.

#### Accelerometer, GPS, travel diary, and LifeLog

Participants were enrolled for one week and wore a GPS data logger (GlobalSat DG-100; New Taipei City, Taiwan) to record geospatial locations, and a hip-mounted accelerometer (ActiGraph GT1M; Pensacola, FL, USA) to measure movement. Participants also recorded place names, addresses, times of arrival and departure, activities at each place, and travel mode from place to place in a travel diary during the same days in which they wore the GPS and accelerometer.

Using the time-stamp as a common identifier, accelerometer, GPS, and travel diary data were combined into a “LifeLog”, which is an individual-level master table for each participant. One record in the LifeLog represents a 30-s time-stamp with corresponding accelerometer counts, GPS latitude, longitude, and speed, and travel diary place or trip characteristics. Detailed methods and description for creating the LifeLog were documented in previous studies [12, 19].

#### Processing GPS and accelerometer data with PALMS and merging it with the LifeLog

PALMS allows researchers to specify analytical parameter settings for identifying trips and places. In this study, PALMS version R4 default parameters were used to process GPS and accelerometer data from the sixty subjects. PALMS calculates the distance and speed between sequential GPS points. Subsets of GPS points were flagged as being members of trips if they spanned ≥ 100 m within an interval of 180 s. Trips with a 90th percentile speed of ≥ 25 km h^−1^ were categorized as driving. Trips with a 90th percentile speed ≥ 10 km h^−1^ and < 25 km h^−1^ were classified as bicycling. Finally, trips with a 90th percentile speed ≥ 1 km h^−1^ and < 10 km h^−1^ were identified as walking. Stationary places between trips were identified as having a duration of ≥ 300 s with GPS points within a 30 m radius.

To generate complete tables (i.e. with one GPS measurement per accelerometry epoch), PALMS imputes GPS points that were missing due to signal loss by duplicating records at the mean coordinate of the 20 records collected before signal loss (see Meseck et al. [38] for detailed information on imputation of GPS data). For the 60 participants over the one-week measurement period, there were 1,712,721 measured records, and PALMS imputed 177,779 GPS records (10.4%). Output from PALMS included original variables (XY coordinate, speed, accelerometry counts), as well as calculated trip and place variables. For comparison with the LifeLog data, PALMS was configured to use 30 s time-stamps.

Results from PALMS were exported as CSV format files. We linked PALMS trip and place information with the LifeLog using subject ID, date, and time stamp as common identifiers. Incomplete travel diary records (e.g., missing trip start or end time, 0.01%), trips with an unspecified travel mode (0.3%), trips taken on ferries (0.002%), and tours (trips recorded with the same start and end location, 0.3%) were removed from the data set. Consequently, 987,550 observations of GPS points with complete PALMS and LifeLog data (99.4% of merged data) remained.

### GPS point level analyses

PALMS and travel diary data were first compared at the GPS point level to determine the amount of agreement between PALMS and travel diary identified trips, places, and travel mode. For trip/place identification comparison, two variables were created for each observation: one indicating whether the PALMS algorithm classified the GPS point as part of a trip or place, and another indicating whether the travel diary classified the observation as part of a trip or place. These two variables were used in a 2 × 2 contingency table to calculate trip classification agreement at the GPS point level.

For travel mode identification comparison, three variables were created for each observation. PALMS classifies trips into one of three travel mode categories (pedestrian, bicycle, or vehicle), whereas the travel diary included 14 options: 1 (auto/truck/van), 2 (carpool/vanpool), 3 (bus), 4 (light rail), 5 (monorail/trolley), 6 (heavy rail), 7 (dial-a-ride/paratransit), 8 (school bus), 9 (ferry), 10 (taxi/shuttle bus/limousine), 11 (motorcycle/moped), 12 (bicycle), 13 (walk), 14 (airplane). For this analysis, we recoded the ten motor vehicle-based travel modes in the travel diary (1–8, 10, 11) as vehicle. These three categories were used in a 3 × 3 contingency table to calculate travel mode classification agreement.

The merged GPS point level data were examined at the subject level to compare the number of trips and places for each participant *i*. The average difference (*D*) between PALMS and travel diary was calculated in Eq. (1), where $x_{{P_{i} }}$, $x_{{TD_{i} }}$ are the number of trips (places) in PALMS *i* and travel diary *i*, and *n* is the number of study participants.

1 $$\begin{array}{*{20}c} {D = \frac{{\mathop \sum \nolimits_{i = 1}^{n} \left( {x_{{P_{i} }} - x_{{TD_{i} }} } \right)}}{n}} \\ \end{array}$$

### Trip level analyses

Trips recorded in the travel diary were used as the unit of analysis in a trip-level data set. PALMS and travel diary data were first tested for matching based on the overall number of trips, and more stringently, based on the temporal overlap between trips identified in these two sources. Each travel diary recorded trip had a unique trip number, together with information about starting time, ending time, duration of a trip, and travel mode. Trips from PALMS also had unique trip IDs and were matched with travel diary trips based on subject ID, date, and time.

Trips from PALMS and the travel diary were considered to match if they had any temporal overlap. We computed an agreement rate (*AR*_*t*_) as the proportion of trips recorded in travel diaries that were matched to PALMS trips (Eq. 2), where *m*_*t*_ is the total number of matching trips, and $x_{{TD_{t} }}$ is the total number of trips from travel diaries.

2 $$\begin{array}{*{20}c} {AR_{t} = \frac{{m_{t} }}{{x_{{TD_{t} }} }} \times 100} \\ \end{array}$$

Next, because times reported in travel logs may not match device times precisely, we performed a sensitivity analysis by repeating the matching analysis using different proportions of overlap time. Specifically, we tested > 0%, > 25%, > 50%, > 75% and 100% of PALMS trip duration overlapping with travel diary trip duration. We computed the percentage of overlapped time (*OT*) between each PALMS and travel diary trip in Eq. (3), where *r* is duration of temporal overlap between PALMS and travel diary, and *t*_*TD*_ is time duration of a trip recorded in travel diary.

3 $$\begin{array}{*{20}c} {OT = \frac{r}{{t_{TD} }} \times 100} \\ \end{array}$$

Lastly, trip-level OT data were aggregated to subject-level agreement rates (*AR*_*i*_) using the five OT cut-off points of > 0%, > 25%, > 50%, > 75% and 100%, as an assessment of PALMS subject-level performance in Eq. (4), where *m*_*i*_ is the total number of matching trips from participant *i*.

4 $$\begin{array}{*{20}c} {AR_{i} = \frac{{m_{i} }}{{x_{{TD_{i} }} }} \times 100} \\ \end{array}$$

To investigate factors associated with differential PALMS subject-level performance across the sixty participants, personal characteristics including primary travel mode and socioeconomic factors (e.g., age, sex, race, education, household income, children, car ownership) were taken into account. Two types of response variables were used in statistical modeling. First, subject-level agreement rates were used as a response variable. Second, since subject-level agreement rate is the proportion of correctly identified trips recorded in travel diary, we also investigated the relationship between the number of matching trips and participants’ personal characteristics. Ordinary least squares and linear mixed effects models were used for the first response variable; negative binomial and mixed effects negative binomial models were applied for the second response variable.

## Results

### GPS point level performance

#### Classification of GPS points in PALMS and travel diary

PALMS classified 56.0% of trip observations in the travel diary as trips and 94.7% of place observations in travel diary as places (Table 1). The level of agreement between the two measures was also assessed by the inter-rater reliability test. Cohen’s kappa statistic was 0.463 (*p* value < 0.01) between PALMS and travel diary.

**Table 1:** Trip and place classification agreement at the GPS point level

	Travel diary (LifeLog)			
	Trip		Place	
	GPS point counts	(%)	GPS point counts	(%)
*PALMS*				
Trip	41,495	(56.0%)	48,016	(5.3%)
Place	32,613	(44.0%)	860,970	(94.7%)
Total	74,108		908,986	

Table 2 shows the travel mode classification results across all participants at the GPS point level. The overall GPS point level agreement rate for travel mode match between PALMS and travel diary was 77.4%. Cohen’s kappa statistic was 0.484 (*p* value < 0.01). The agreement rate between PALMS and travel diary observations was higher for vehicle versus bicycling and walking.

**Table 2:** Travel mode classification at the GPS point level

	Travel diary (LifeLog)					
	Vehicle		Bicycle		Walking	
	GPS point counts	(%)	GPS point counts	(%)	GPS point counts	(%)
*PALMS*						
Vehicle	25,963	(84.5%)	1024	(45.6%)	2271	(26.6%)
Bicycle	2916	(9.5%)	1202	(53.5%)	1299	(15.2%)
Walking	1835	(6.0%)	20	(0.9%)	4965	(58.2%)
Total	30,714		2246		8535	

#### Comparing trips and places identification by individual participant

**Fig. 1:**
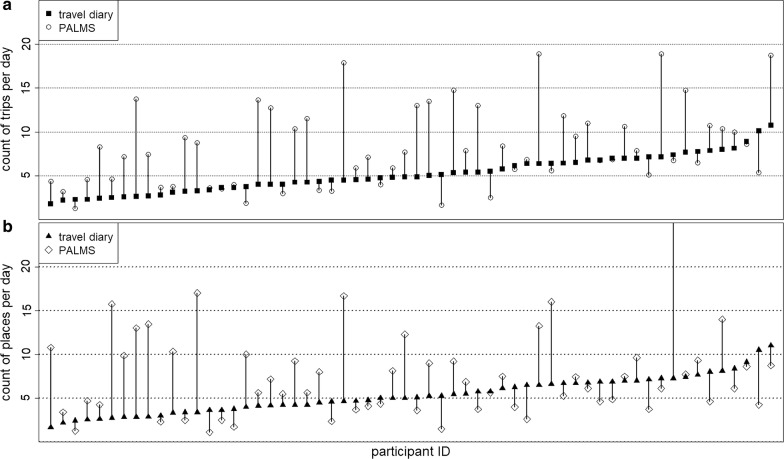
Count of trips (**a**) and places (**b**) per day by study participant

Figure 1 shows the difference in the number of places and trips per day extracted from the travel diary and from PALMS by participant. Differences did not appear to be consistent across participants. PALMS found an equal or greater number of trips for 71.7% of the sample and fewer trips for 28.3% of the sample relative to the travel diary (Fig. 1a). Also, PALMS identified an equal or greater number of places for 55% of the sample, and found fewer places among 45% of the sample (Fig. 1b). On average, PALMS identified 3.1 more trips and 2.1 more places per day by participant during the seven consecutive days than were found in the travel diary.

#### Comparing travel diary and PALMS data for one day of activity from two subjects

**Fig. 2:**
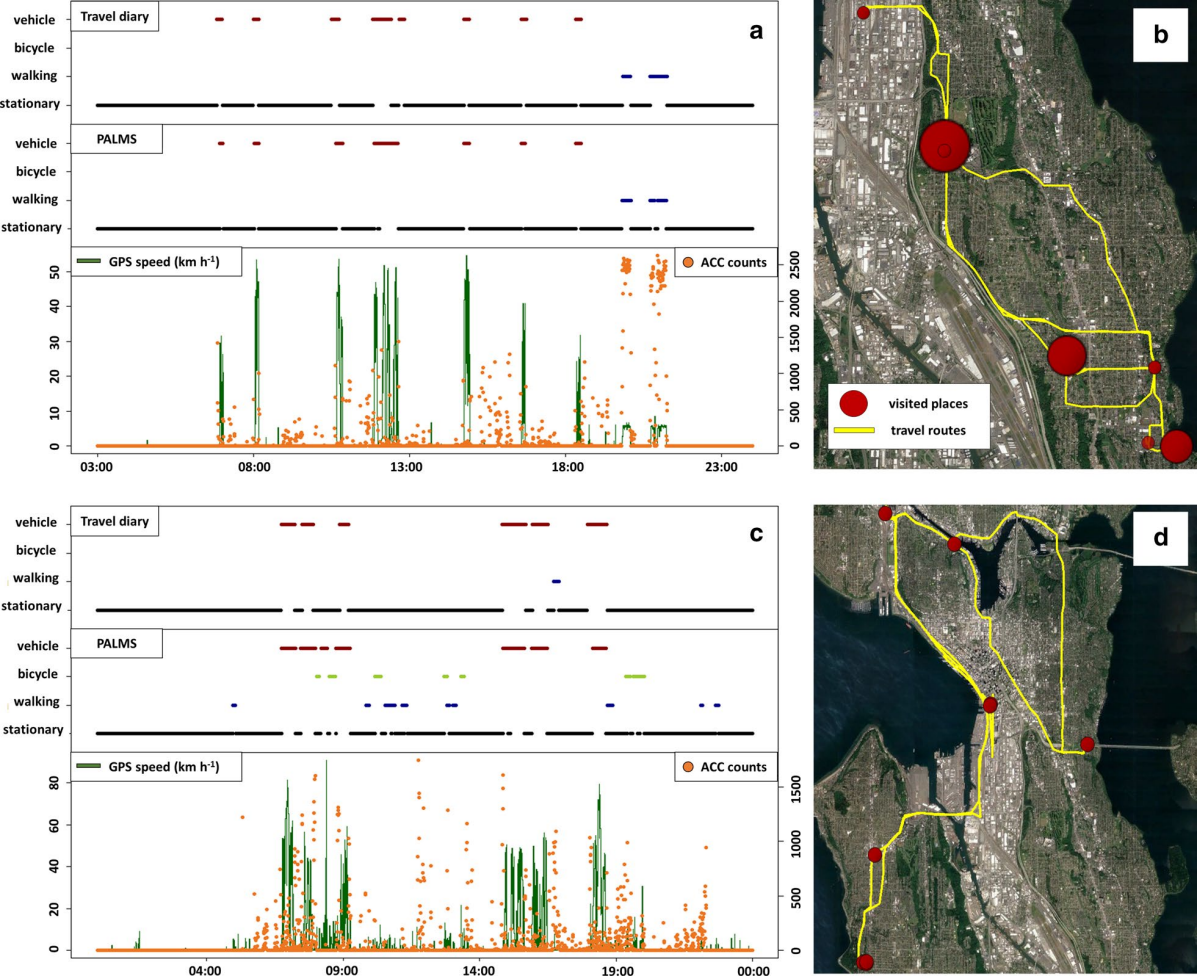
**a**, **c** show an example of high and low agreement between travel diary and PALMS outcomes, respectively. **b**, **d** represent places and travel routes identified by PALMS for each case. A bigger circle means high frequency of visit in the map

Visualization of travel diary and PALMS outcomes with corresponding maps provides some insight into the observed discrepancies. Figure 2 shows travel diary and PALMS data for one day of activity from two subjects. The upper and lower panels show an example of high and low agreement between travel diary and PALMS outcomes, respectively.

In the high agreement case (Fig. 2a, b), eight vehicular trips and two walking trips were reported in the travel diary; PALMS identified seven vehicular trips and two walking trips for this day for this subject. Based on these visuals, we could conclude that both travel diary and PALMS travel behavior identification algorithm worked well for this subject for this day.

In the low agreement case (Fig. 2c, d), the travel diary records included six vehicular trips and one walking trip. However, PALMS identified eight vehicular trips, seven bicycling trips, and nine walking trips within the same time period. Considering GPS speed and accelerometer count patterns between 09:00 and 14:00, and 19:00 to 00:00, this subject may have neglected recording some walking trips in travel diary. On the other hand, a single vehicular trip might have been identified as multiple vehicular and bicycling trips in PALMS. It is possible that vehicular trips with low speed were identified as bicycling trips in PALMS.

### Trip level performance

#### Global performance

There were 2100 trips in the travel diaries of the sixty subjects across all modes. The days on which these trips were recorded had 2483 trips across all modes derived from PALMS. 1233 trips from the travel diary were identified by PALMS as trips (regardless of mode) (58.7%), 598 (28.5%) travel diary trips were not matched to a PALMS trip, and 269 (12.8%) travel diary trips were split into more than one trip in PALMS. Of the 598 travel diary trips that were not captured by PALMS, 56% were reported as vehicular trips, 41.1% were reported as walking, and the remaining 2.9% were reported as bicycle trips.

**Fig. 3:**
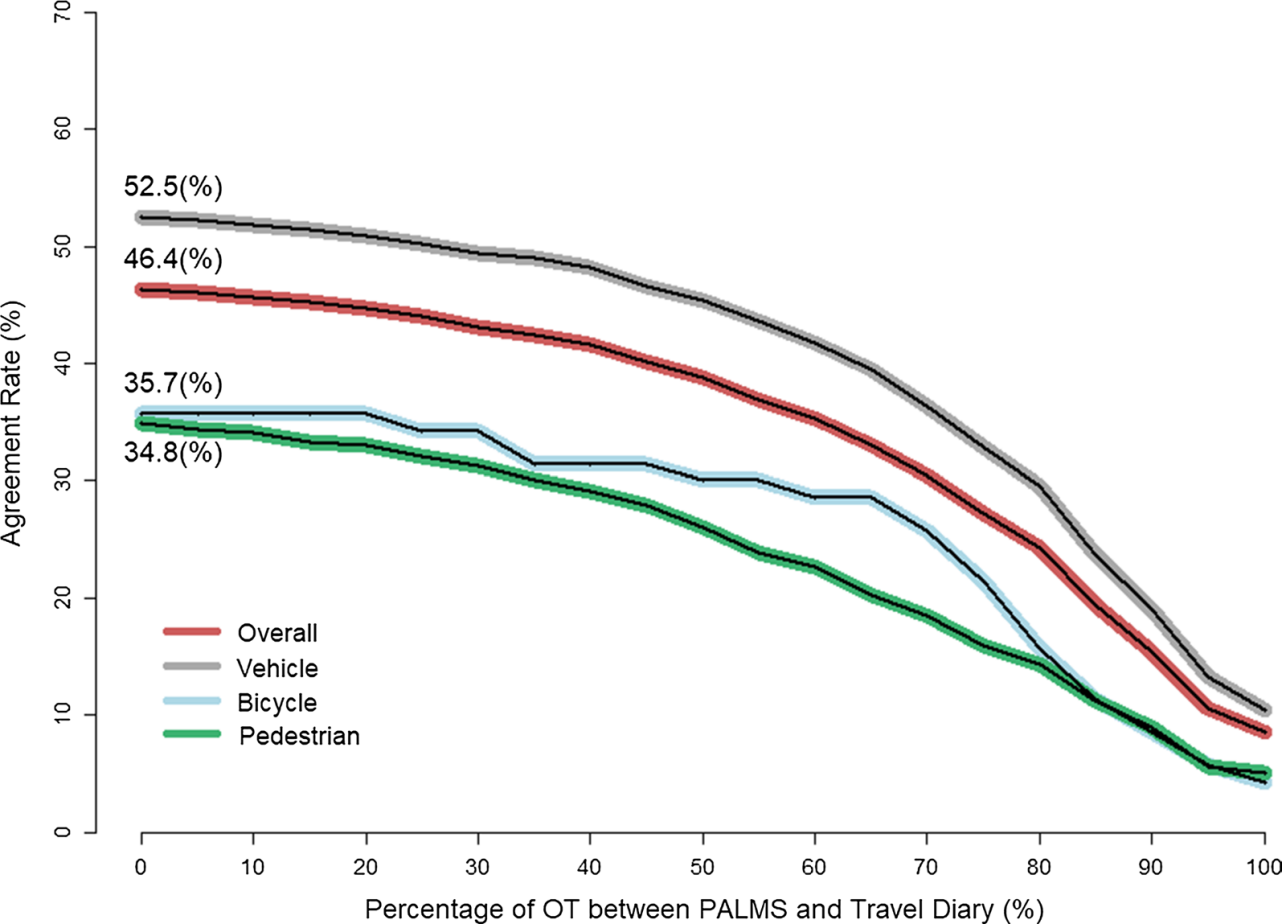
Agreement rate in the different modes by percentage of overlapped time (OT)

Figure 3 shows the agreement rates (*AR*_*t*_) by varying the percentage of overlap time (OT) (0–100%) between PALMS and the travel diary. As expected, agreement rates were greater for lower values of OT percentage. The overall agreement rate (OT > 0%; where there was any temporal overlap between PALMS and travel diary trips) across the three travel modes was 46.4%. The agreement rate for vehicle, bicycle and pedestrian trips was 52.5%, 35.7%, and 34.8%, respectively.

#### Subject level performance

To assess subject-level correspondence between PALMS and travel diary trip identification, we calculated agreement rates for each subject (*AR*_*i*_). The total number of correctly identified trips per subject was first calculated by applying the same methods used in global performance analyses. This number of matched trips was divided by the total number of trips recorded in the subject’s travel diary.

**Fig. 4:**
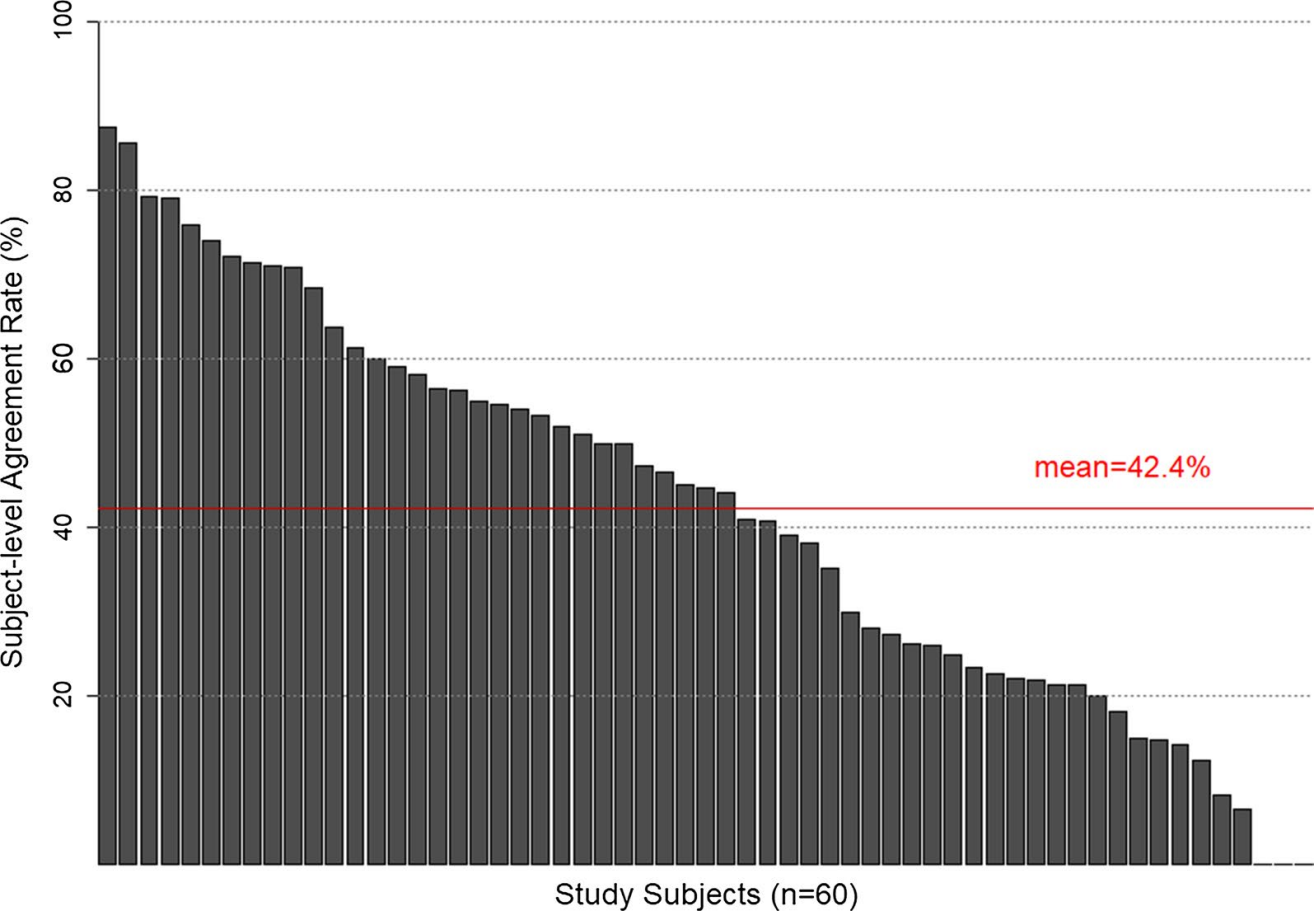
Subject-level agreement rates (OT > 0%)

Figure 4 shows inter-subject variation in agreement rates based on having any OT. Of the 60 subjects, 10 (17%) had an agreement rate that was lower than 20%. Among these subjects, three had 0% agreement rates. Proportions of subjects that had an agreement rate of 20–40%, 40–60%, 60–80%, 80–100% were 27.1%, 32.2%, 20.3% and 3.4%, respectively. Based on these subject-level agreement rates, the mean agreement rate was calculated as 42.4% (OT > 0%).

#### Statistical modeling for PALMS subject-level performance

Four statistical models were applied to investigate variations in subject-level performance (OT > 0%) (Table 3). Models I and II show OLS and negative binomial modeling results. Also, since all subjects were clustered in three primary travel modes, we applied mixed-effects models to reflect hierarchical data structure in models III and IV. The average subject-level agreement rate was 5*9*.4%, 20.5%, 48.2% for driver, transit user, and walker groups, respectively.

**Table 3:** Statistical modeling results

Response variable (PALMS subject-level performance; OT > 0%)	Model I (OLS)			Model II (Negative binomial)			Model III (Linear mixed effects)			Model IV (Mixed effects negative binomial)		
	Subject-level agreement rate			# of Matching trips			Subject-level agreement rate			# of Matching trips		
	*B*	*CI*	*p*	*IRR*	*CI*	*p*	*B*	*CI*	*p*	*IRR*	*CI*	*p*
							Fixed parts					
(Intercept)	20.78	− 5.15 to 46.72	0.114	0.21	0.11 to 0.40	< 0.001	38.38	10.40 to 66.36	0.007	0.38	0.18 to 0.80	0.011
Age	0.07	− 0.37 to 0.50	0.758	1	0.99 to 1.01	0.961	0.07	− 0.32 to 0.47	0.719	1	0.99 to 1.01	0.907
Sex (ref. female)	− 3.46	− 12.85 to 5.93	0.462	0.95	0.79 to 1.15	0.622	− 4.56	− 13.04 to 3.92	0.292	0.93	0.76 to 1.13	0.469
Race (ref. non-white)	− 7.78	− 19.65 to 4.08	0.193	0.87	0.67 to 1.14	0.311	− 7.73	− 18.53 to 3.08	0.161	0.89	0.67 to 1.17	0.394
Education (ref. < college grad)	− 3.46	− 15.54 to 8.61	0.567	0.95	0.73 to 1.24	0.71	− 1.9	− 12.78 to 8.98	0.732	0.98	0.75 to 1.29	0.909
Household Income (ref. ≤ $50 k)	− 2.92	− 15.49 to 9.66	0.643	0.92	0.72 to 1.19	0.531	− 1.93	− 13.34 to 9.49	0.741	0.94	0.72 to 1.22	0.628
Children (ref. no children)	1.51	− 12.36 to 15.38	0.828	1.02	0.77 to 1.36	0.869	2	− 10.63 to 14.63	0.757	1.02	0.76 to 1.37	0.891
Car ownership (ref. no car)	15.01^**^	1.61 to 28.41	0.029	1.34^*^	0.99 to 1.82	0.057	16.54^***^	4.47 to 28.61	0.007	1.41^**^	1.03 to 1.93	0.034
Primary travel mode (ref. *transit user*)							Random parts					
Driver	32.96^***^	16.78 to 49.14	< 0.001	2.62^***^	1.74 to 3.97	< 0.001	σ^2^ = 219.324			–		
Walker	26.87^***^	12.38 to 41.36	< 0.001	2.34^***^	1.60 to 3.45	< 0.001	τ_00_ = 154.055			τ_00_ = 0.138		
–	–	–	–	–	–	–	N = 3			N = 3		
–	–	–	–	–	–	–	ICC = 0.413			ICC = 0.056		
Observations	57			57			57			57		
R^2^/adj. R^2^	0.581/0.501			–			R^2^/Ω_0_^2^ = 0.578/0.577			Deviance = 51.43		

* *p* < 0.1; ** *p* < 0.05; *** *p* < 0.01

Car ownership was statistically significant and positively associated with PALMS subject-level performance in all models with 95% confidence interval except for model II (*p* value = 0.057). Primary travel mode was also statistically significant in models I and II. Being in the driver or walker group (with the transit user group as the reference category) was related to higher performance in PALMS. Primary travel mode was also used as a random intercept in mixed-effects models (III, IV). The intra-class correlation coefficient (ICC) was higher in model III. Other personal characteristics were not related to PALMS subject-level performance.

**Fig. 5:**
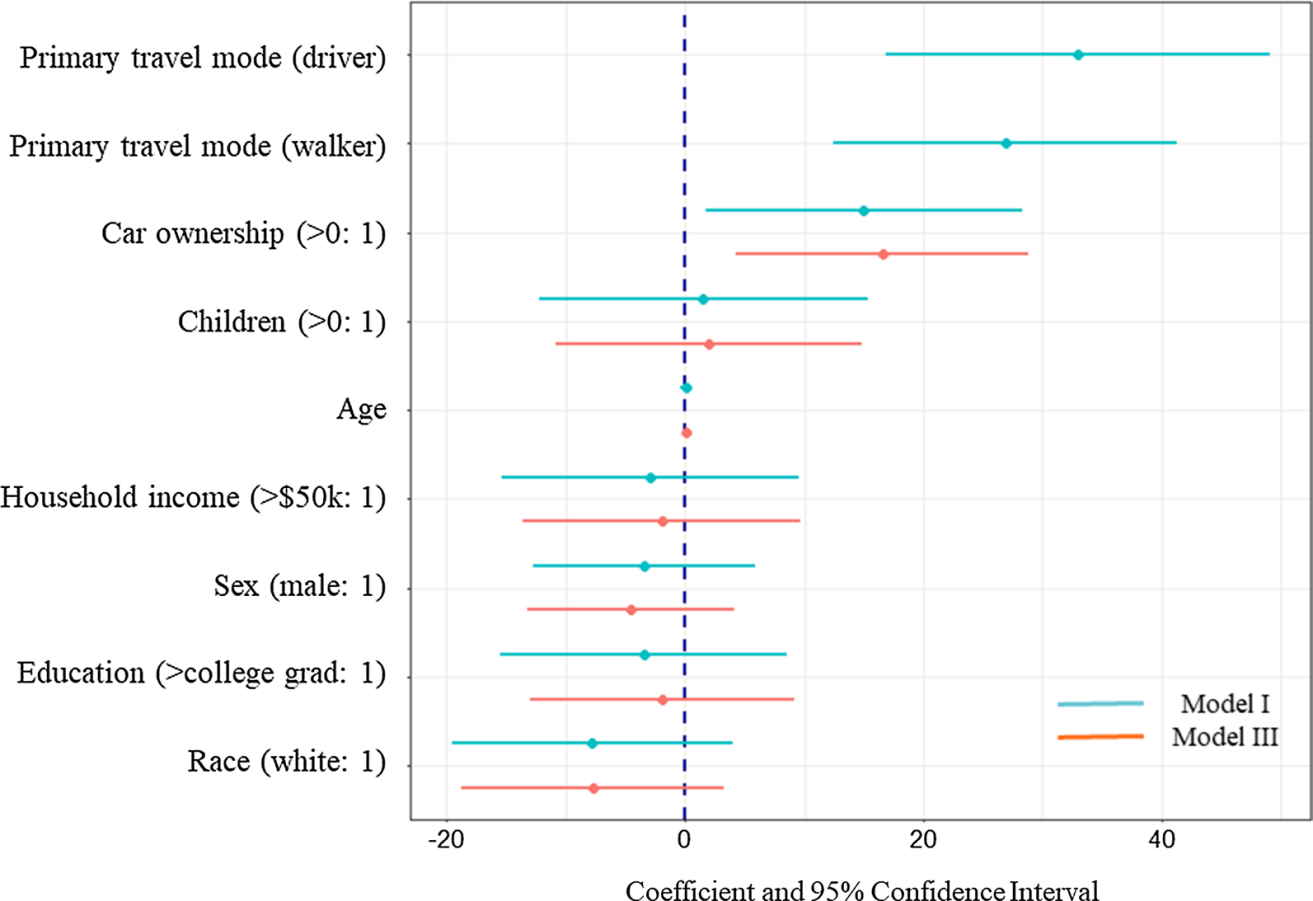
Coefficient and confidence interval plot for subject-level agreement rate regression (Model I and III)

**Fig. 6:**
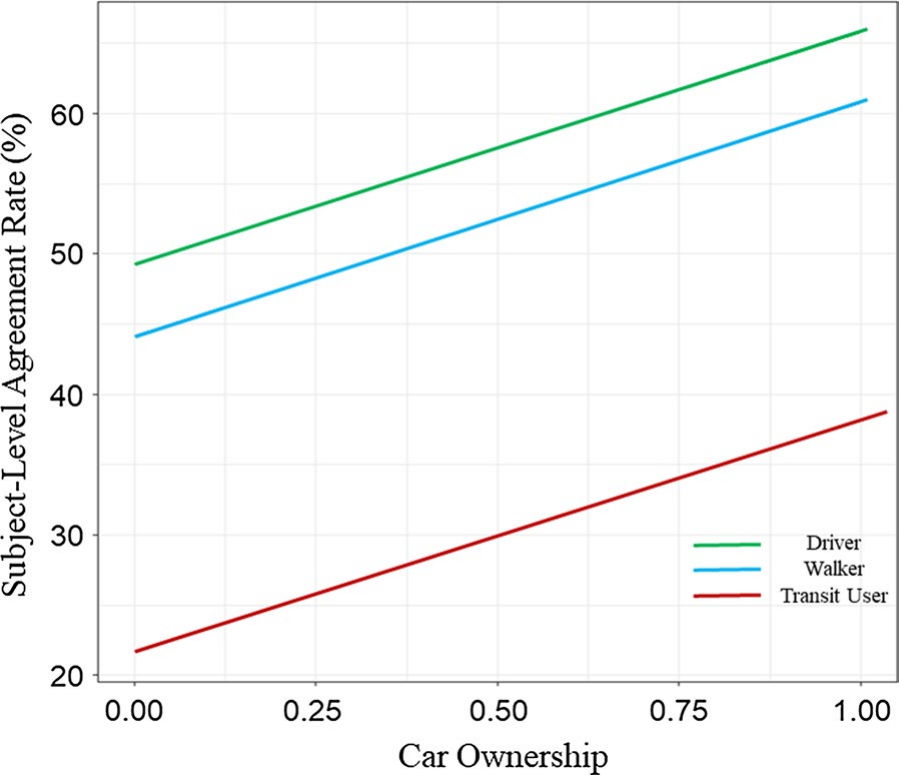
The effect of car ownership on subject-level agreement rate by primary travel mode

The hierarchical data structure was better explained in the linear mixed-effects model (model III), which is summarized in Fig. 5. Bars that do not span 0 (e.g., travel mode and car ownership) indicate a significant relationship. For comparison, OLS model (model I) results were also included in the figure. With car ownership being the only statistically significant variable in model III, it was visualized using random intercepts by setting all other covariates to zero in Fig. 6, which shows both of random intercepts and the fixed coefficient of car ownership. Agreement rates increased with car ownership for all subjects, but transit users had significantly lower agreement rates compared to the high drivers and high walkers.

## Discussion

This study compared PALMS place, trip, and travel mode identification results with travel diary data from sixty adults living in a mid-sized US metropolitan area, the greater Seattle area, and selected for having different primary travel modes. Compared to previous GPS algorithm assessment studies [5, 8, 10, 25], the study used a larger sample and examined travel mode agreement among 2100 recorded trips with 987,500 GPS point observations. The two data sets offered two levels of analysis.

At the GPS point level analysis, PALMS trip and place classification agreement rates were quite different at 56.0% and 94.7%, respectively. The place identification agreement rate was similar to that of a study which compared PALMS outcomes with SenseCam images sampled every minute (93.4%) [25], but the trip identification agreement rate was lower (88.5%) [25]. Since the present study used the self-reported travel diary as reference data, the results cannot be directly compared with objectively captured photographic data. However, the difference between the two studies suggest that diary data for places is more precisely recorded than it is for trips. The selected population mixing primarily drivers, transit users, and walkers could also explain the different results.

Agreement rates varied significantly by travel mode. While the rate was high at 77.4% for all observations, they were lower for bicycle (53.5%) and walking trips (58.2%) compared to vehicular trips (84.5%) at the GPS point level. Notably, 45.6% of bicycling trip GPS points recorded in the travel diary were classified by PALMS as driving. Distinguishing between driving and bicycling can be difficult due to primary reliance on speed recorded in GPS data. In PALMS, the cut-off speed used for differentiating driving from bicycling was 25 km h^−1^. Results would likely be different using higher or lower cut-off speeds. In addition, the number of observations for bicycling trips in our sample (2246 GPS points from 70 trips) was relatively small compared to other travel modes (vehicle: 30,714 GPS points from 1367 trips; walking: 8535 GPS points from 663 trips). It is also likely that distinguishing between motor vehicle travel and bicycling is more challenging in higher traffic and congested areas due to lower vehicular speeds. The use of ancillary data, such as measured heart rate, which was not available for this study, would enhance the performance of PALMS for separating bicycle trips from driving [39].

The difficulties in identifying trips and travel modes were also confirmed through individual participant level analyses. PALMS identified an average of 23.9 more trips per subject than were recorded in the travel diary, and 71.7% of subjects had more trips identified by PALMS than were recorded in the travel diary. Disparities in trip counts were much higher than the discrepancies seen for PALMS place identification algorithm results (16.3 more places per subject on average; 55% of subjects with more places in PALMS versus the travel diary). Visualizing mapped data from two subjects helped points to known limitations in travel diary data, which are prone to errors and omissions in recorded trips [5, 15, 17]. Evidence of unrecorded trips can be found in GPS and accelerometer data. Also, many single trips as recorded in the travel diary were split into multiple trips by PALMS. However, these errors could be minimized by careful selection of parameter settings in PALMS and by varying tolerances for acceptable short-duration stops within trips.

Since the GPS point level analyses revealed some limitations, we reconstructed our data at the trip level and compared PALMS outcomes with the travel diary. We took a novel approach of reporting global and subject-level performance. In the global performance analyses, we found that 12.8% of trips recorded in the travel diary were split into multiple trips in PALMS. This result confirmed one of our speculations about why PALMS identified more trips than travel diary records. For trip level analyses, we took the most conservative perspective by excluding these divided trips from correctly identified trip group.

In the global performance analyses, we advanced the agreement analyses by introducing the percentage of OT between PALMS and travel diary. In the analyses, the overall agreement rate was lower (46.4%, OT > 0%) than the results from GPS point level analyses (77.4%). Since we used strict rules by removing divided trips from the correctly identified trip group, many GPS points previously labeled as matched points were classified as unmatched trips in this level of analysis.

The agreement rate between PALMS and travel diary travel modes at the trip level was highest for vehicular trips (52.5%, OT > 0%), consistent with GPS point level analysis results. Bicycling had the second highest agreement rate (35.7%, OT > 0%), with walking trip agreement rate being slightly lower (34.8%, OT > 0%). At the GPS point level, agreement for walking was higher than for bicycling (58.2% vs. 53.5%). However, the agreement rate for vehicular trips was much higher (84.5%). This discrepancy is likely due to the parameters for speed used to identify walking, bicycling, driving, which were specified as 1–10 km h^−1^, 10–25 km h^−1^, and ≥ 25 km h^−1^, respectively. Walking and bicycling usually do not exceed 25 km h^−1^. However, motor vehicles often drive at 25 km h^−1^ for extended intervals, particularly in urban areas with high traffic congestion. The overlap in actual speeds increases the difficulty in differentiating different travel modes. The agreement rate was also highest in vehicular trips in a previous study that assessed PALMS [25].

In the subject-level performance analyses, the overall agreement rate (42.4%, OT > 0%) was slightly lower than in the global performance analyses (46.4%, OT > 0%). Subject-level agreement rates were highly variable across subjects, with a range of 0–87.5%. To further investigate this discrepancy, we included primary travel mode and socioeconomic information as covariates in subject-level models.

Primary travel mode was found to be the strongest explanatory variable examined for the discrepancy between PALMS and travel diary travel modes. Subject-level agreement rates were highest among those identified primarily as drivers and lowest among primarily public transit users. A previous study showed that transit users have more walking trips than non-users, which will ostensibly affect their individual-level agreement rates [40]. Also, transit trips have spatial and temporal characteristics that may make them more difficult to identify: they have more short-duration stops in designated areas than driving trips; public transit vehicles mostly run along arterial roads with stop-and-go traffic signals and congestion; and they may be more subjected to the urban canyon effects than driving trips, which can lower the quality of GPS data [5]. Owning a car was also found to be significantly related to higher subject-level agreement rates, in line with the global performance analyses results which showed that PALMS can best detect vehicular trips. This was notable because car ownership was significant in these model even after accounting for the primary mode grouping (driver, transit user, walker) and the high level of vehicle ownership in the sample.

Overall, the results from this study showed lower agreement rates than previous studies [8, 25] because we took a more conservative approach and applied strict rules to assess the algorithm. However, the detailed investigation methods will provide new information and help developers and users to improve their future algorithms. Our study also has limitations. First, there is currently no gold standard criterion measure for travel behavior identification, and it is likely that both the PALMS algorithm and the travel dairy had errors and omissions. Future studies could be aided by the use of additional objective reference data (e.g., heart rate monitoring for walking and bicycling) to evaluate the algorithm. Second, we used default parameter settings in PALMS for our analyses. Agreement rates for active travel modes may be improved through careful selection of PALMS parameters.

Third, the TRAC study participants were sampled from the greater Seattle area, which contains pockets of high-density development and experiences severe commute-time traffic congestion on expressways and on arterial roads. The quality of individual-level GPS data might be better in suburban and rural areas where the configuration of built environments is less complex and traffic congestion is less frequent. Fourth, the TRAC sample did not include people with physical disabilities, which affect the travel mode choice. Still, anyone can suffer temporary physical or mental disabilities during the study period. Future studies may consider these confounders to better explain the subject-level performance of PALMS. Fifth, although the descriptive statistics of study participants showed that the sample is not biased, there is a possibility of self-selection in survey samples. To mitigate the problem, we applied a stratified random sampling method to proportionally include under-represented travel mode groups (e.g. public transit user). The performance of PALMS needs to be tested in different geographic areas where a higher proportion of the population uses transit or non-motorized modes. Such areas will likely also have different built environments and socio-demographics. Lastly, compared to previous studies, our larger sample still did not provide ample power for statistical modeling. Future studies may include a larger population.

## Conclusions

The use of objectively measured data from such devices as GPS and accelerometers has dramatically changed research methods in the transportation planning and public health fields during the last fifteen years. Automated algorithms have enabled researchers to process large data sets and to identify fine-scaled and active travel behaviors in less time and with fewer errors. This study investigated the performance of PALMS, a widely used travel behavior identification system.

For a thorough investigation, we used multiple methods to compare PALMS outcomes with travel diary data. In analyses at the GPS point level, PALMS showed moderate agreement for trips and travel modes. Trip level analyses yielded moderate agreement rates for vehicular trips, but walking and bicycling trips had lower agreement rates. At the subject level, agreement rates were lower for the public transit user group than for the driver and the walker groups. It appears that trips taken by individuals who are primarily transit users are more difficult to identify: transit users take more walking trips and their many transit trips occur in densely developed areas with more traffic congestion, stop-and-go traffic flow, and lower quality GPS data due to poor reception in canyon-like settings.

PALMS appears to perform well in identifying vehicular trips and corresponding travel routes, indicating that the algorithm can help reduce the burden of processing GPS data into travel behavior data. To enhance the overall performance of the algorithm, further efforts are needed to identify non-motorized travel modes. The use of shorter measurement intervals for GPS and accelerometer data, as well as adjustments to speed thresholds suggested as defaults in the algorithm, may improve differentiation between walking and other modes. Ancillary data sources, such as heart rate monitoring, may help differentiate active (walking, bicycling) and passive (vehicular) modes. Efforts to discriminate between public transit and automobile trips will further expand the usefulness of PALMS. Geographic information systems (GIS) data already in existence, such as transit routes, stops, and stations may play a strong role. Finally, systematic evaluation of different parameter settings in PALMS could lead to better detection of places versus trips and short-duration stops within trips.

PALMS is a promising tool that serves to link data capturing health and transportation behaviors. Its performance in detecting active travel modes will be improved with further testing of the different parameter settings. Also, testing and evaluating PALMS internationally in a range of urbanized areas where travel modes other than driving are common will broaden the applicability of the algorithm.
